# Pricing for a basket of LCDS under fuzzy environments

**DOI:** 10.1186/s40064-016-3420-x

**Published:** 2016-10-07

**Authors:** Liang Wu, Jie-fang Liu, Jun-tao Wang, Ya-ming Zhuang

**Affiliations:** 1 Department of Mathematics, Henan Institute of Science and Technology, Xinxiang, 453003 Henan People’s Republic of China; 2 School of Economics and Management, Southeast University, Nanjing, 211189 Jiangsu People’s Republic of China

**Keywords:** Prepayment risk, One-factor copula function, Fuzziness and hesitation, First default pricing of a basket of LCDS

## Abstract

This paper looks at both the prepayment risks of housing mortgage loan credit default swaps (LCDS) as well as the fuzziness and hesitation of investors as regards prepayments by borrowers. It further discusses the first default pricing of a basket of LCDS in a fuzzy environment by using stochastic analysis and triangular intuition-based fuzzy set theory. Through the ‘fuzzification’ of the sensitivity coefficient in the prepayment intensity, this paper describes the dynamic features of mortgage housing values using the One-factor copula function and concludes with a formula for ‘fuzzy’ pricing the first default of a basket of LCDS. Using analog simulation to analyze the sensitivity of hesitation, we derive a model that considers what the LCDS fair premium is in a fuzzy environment, including a pure random environment. In addition, the model also shows that a suitable pricing range will give investors more flexible choices and make the predictions of the model closer to real market values.

## Background

Data from The People’s Bank of China reveals that, by the end of December 2015, residential mortgages had exceeded 10 trillion CNY; an amount 16 times the 660 billion CNY in residential mortgages that existed at the end of 2001. It is widely known that there are great risks associated with the housing mortgage business when a market is rising; once a bubble forms from overvalued housing, demand may fall with a resulting excess of housing stock. Defaults in mortgage repayments caused by the sharp falls in housing values cannot be avoided and a financial crisis caused by a shortage, or even collapse, of bank liquidity may follow. The sub-prime debt crisis in the US in 2007 and the sharp fall in housing prices in Hangzhou, China in February 2014, are examples.

As mentioned above, residential mortgages are a core business of most banks in China, and it goes without saying that evaluation of credit risk is of paramount importance to the safe operation and success of lending. Credit risk is defined as the risk of default when a borrower is reluctant, or unable, to repay principle and interest on a loan. It follows that the financial health of a bank depends on the dispersion of credit risk throughout the banking system and transferring risk to second or even third level investors is a powerful way to reduce the severity of risk and avoid markets being impacted. How to effectively manage housing mortgage credit risk is, and will be, the object of intense study.

Recently, credit derivatives have become an important means of managing international bank and financial market credit risk. Loan credit default swaps (LCDS), first promoted in America and subsequently in Europe by the International Swap and Derivatives Association (ISDA), by 2006 had become a powerful financial derivative instrument in the dispersal, transfer and hedging of mortgage loan credit risk. Credit Loan Default Swaps have the particularity that their reference entity is the mortgage loan. It is widely believed that credit derivatives to some extent were to blame for the 2007 sub-prime debt crisis in the United States. They did, however, also serve to objectively isolate the financial institutions, especially in the banking system, from risks, thereby freeing many banks from possible default bankruptcies. Credit derivatives should be carefully developed to create optimal conditions to lighten the impact of loan risk for banks. We should learn from the sub-prime debt crisis in the United States to enhance supervision in the credit derivatives market, we should research thoroughly the pricing theory of credit derivatives under conditions of incomplete information to achieve optimal pricing, and we should build a more effective model to better resemble the real financial market.

There are two differences between LCDS and standard credit default swaps. 1) A borrower may prepay a mortgage and there is a risk that with LCDS the contract will end ahead of time; this is different from a common reference asset with a certain due date. 2) A common reference asset is unsecured while a mortgage is secured and thus may make a higher recovery amount in the case of LCDS defaults, higher than with standard contracts. At the present time, research on LCDS pricing mainly uses structural models to determine LCDS pricing and considers changes in the value of a company and mortgage asset (Wu and Liang 2011; Rong et al. 2012; Liang et al. 2013; Zhou and Liang 2010; Wu 2012; Liang and Wang 2012; Wang et al. 2012). Some literature (Wu and Liang 2011; Rong et al. 2012) takes uni-nominal LCDS pricing and counter-party risk into account while other literature (Liang et al. 2013; Zhou and Liang 2010; Wu 2012; Liang and Wang 2012; Wang et al. 2012) discusses a basket of LCDS pricing using a reduced-form model. All the above research assumes the prepayment risk of a LCDS reference loan to be either a constant or a random process. However, the motivations of borrowers to prepay a mortgage are somewhat complex. These motivations include both market factors and the profile and personal characteristics of borrowers, for example, market interest rate changes or changes of the financial condition of borrowers. This results in a certain fuzziness and hesitation for investors with respect to any prepayment by borrowers and eventually influences the price of LCDS. Hence, by using a reduced-form model to represent prepayment risk and triangle intuition-based fuzzy numbers to describe the fuzziness and hesitation of the sensitivity coefficient in the model, we can make a pricing model that is closer to real market situations. All kinds of fuzzy information exists in the pricing of financial products as there are market uncertainties which cannot be described by Probability Theory. One of the essential factors of financial product pricing is the associated and mutually non-substitutable relationship with random process (related concepts can be seen in reference Wu and Zhuang 2015). There is some literature concerning option pricing in a fuzzy random environment (Ma et al. 2012; Simonelli 2001; Yoshida 2003; Wu 2004; Xu et al. 2009; Zhang et al. 2010, 2013) and the application of uncertain information in other fields (Jiang et al. 2016a, b; Deng 2015, 2017; Ning et al. 2016; Akyar 2016; Wang et al. 2014). There is, however, little research into the pricing of credit derivatives when a fuzzy environment is introduced (Agliardi and Agliardi 2009; Wu and Zhuang 2015; Wu et al. 2016).

This paper discusses the first default pricing of a basket of LCDS contracts with a basket of housing mortgages as the reference asset. This paper: (1) uses the factor Copula function to describe the value of a basket of mortgages because financial markets are changeable and changes in different asset values are relevant; (2) presents the prepayment risk of reference mortgage loans using a reduced-form model and introduces triangular intuition-based fuzzy numbers to describe the fuzziness and hesitation of the sensitivity coefficient; (3) puts forward the first default probability of a reference loan using a structural model and then works out the expression of the first default price of a basket of LCDS, i.e. it uses a mixed model to price the first default of a basket of LCDS.

## Basic concept of triangular intuition based fuzzy numbers

This section reviews some of the basic concepts of triangular intuition based fuzzy numbers, which are closely related to our work.

### **Definition 1**

(Shu et al. 2006; Nan et al. 2010). Let $\tilde{c} = \left\langle {(\underline{c} ,c,\bar{c});\omega_{{\tilde{c}}} ,u_{{\tilde{c}}} } \right\rangle$ be a triangular intuition-based fuzzy number (TIFN) on the real number set R, whose membership function and non-membership function are defined as follows:

$$\mu_{{\tilde{c}}} (x) = \left\{ {\begin{array}{*{20}l} {\frac{{x - \underline{c} }}{{c - \underline{c} }}\omega_{{\tilde{c}}} ,} \hfill & {\underline{c} \le x < c} \hfill \\ {\omega_{{\tilde{c}}} ,} \hfill & {x = c} \hfill \\ {\frac{{\bar{c} - x}}{{\bar{c} - c}}\omega_{{\tilde{c}}} ,} \hfill & {c < x \le \bar{c}} \hfill \\ {0,} \hfill & {x < \underline{c} , \, x > \bar{c}} \hfill \\ \end{array} } \right.\quad {\text{and}}\quad \nu_{{\tilde{c}}} (x) = \left\{ {\begin{array}{*{20}l} {\frac{{c - x + u_{{\tilde{c}}} (x - \underline{c} )}}{{c - \underline{c} }},} \hfill & {\underline{c} \le x < c} \hfill \\ {u_{{\tilde{c}}} ,} \hfill & {x = c} \hfill \\ {\frac{{x - c + u_{{\tilde{c}}} (\bar{c} - x)}}{{\bar{c} - c}},} \hfill & {c < x \le \bar{c}} \hfill \\ {1,} \hfill & {x < \underline{c} , \, x > \bar{c}} \hfill \\ \end{array} } \right.$$

respectively, where the values $\omega_{{\tilde{c}}}$ and $u_{{\tilde{c}}}$ represent the maximum degree of membership and the minimum degree of non-membership, respectively, such that they satisfy the following conditions: $0 \le \omega_{{\tilde{c}}} \le 1,0 \le u_{{\tilde{c}}} \le 1,$ and $0 \le \omega_{{\tilde{c}}} + u_{{\tilde{c}}} \le 1$.

Let $\pi_{{\tilde{c}}} (x) = 1 - \mu_{{\tilde{c}}} - \nu_{{\tilde{c}}}$, which is the intuition-based fuzzy index of an element $x$ in the TIFN $\tilde{c}$. This is the degree of indeterminacy membership of the element $x$ to the TIFN $\tilde{c}$; TIFNs can therefore measure the degree of hesitation when investors make decisions and more objectively, accurately reflect the uncertain information. The literature (Nan et al. 2010) introduces the concept of TIFN cut sets.

### **Definition 2**

(Nan et al. 2010) Let $\tilde{c} = \left\langle {(\underline{c} ,c,\bar{c});\omega_{{\tilde{c}}} ,u_{{\tilde{c}}} } \right\rangle$ be a TIFN, then a $\left\langle {\kappa ,\lambda } \right\rangle$-cut set of $\tilde{c}$ is a crisp subset of R, which is defined as $\tilde{c}_{\lambda }^{\kappa } = \{ x|\mu_{{\tilde{c}}} (x) \ge \kappa ,\nu_{{\tilde{c}}} (x) \le \lambda \}$, where $0 \le \kappa \le \omega_{{\tilde{c}}}$, $u_{{\tilde{c}}} \le \lambda \le 1$, and $0 \le \kappa + \lambda \le 1$.

### **Definition 3**

(Nan et al. 2010) Let $\tilde{c} = \left\langle {(\underline{c} ,c,\bar{c});\omega_{{\tilde{c}}} ,u_{{\tilde{c}}} } \right\rangle$ be a TIFN, then a $k$-cut set of $\tilde{c}$ is a crisp subset of R, which is defined as $\tilde{c}^{\kappa } = \{ x|\mu_{{\tilde{c}}} (x) \ge \kappa \}$, where $0 \le \kappa \le \omega_{{\tilde{c}}}$.

Using the membership function of $\tilde{c}$ and Definition 3, it is easily seen that $\tilde{c}^{\kappa }$ is a closed interval and calculated as follows:

$$\tilde{c}^{\kappa } = \left[ {\tilde{c}_{L}^{\kappa } ,\tilde{c}_{R}^{\kappa } } \right] = [\underline{c} + \kappa (c - \underline{c} )/\omega_{{\tilde{c}}} ,\bar{c} - \kappa (\bar{c} - c)/\omega_{{\tilde{c}}} ].$$

### **Definition 4**

(Nan et al.^2010^) Let $\tilde{c} = \left\langle {(\underline{c} ,c,\bar{c});\omega_{{\tilde{c}}} ,u_{{\tilde{c}}} } \right\rangle$ be a TIFN, then a $\lambda$-cut set of $\tilde{c}$ is a crisp subset of R, which is defined as $\tilde{c}_{\lambda } = \{ x|\nu_{{\tilde{c}}} (x) \le \lambda \}$, where $u_{{\tilde{c}}} \le \lambda \le 1$.

U**s**ing the non-membership function of $\tilde{c}$ and Definition 4, it is easily seen that $\tilde{c}_{\lambda }$ is a closed interval and calculated as follows:

$$\tilde{c}_{\lambda } = [\tilde{c}_{L}^{\lambda } ,\tilde{c}_{R}^{\lambda } ] = [[(1 - \lambda )c + (\lambda - u_{{\tilde{c}}} )\underline{c} ]/(1 - u_{{\tilde{c}}} ),[(1 - \lambda )c + (\lambda - u_{{\tilde{c}}} )\bar{c}]/(1 - u_{{\tilde{c}}} )].$$

### **Definition 5**

(Zhang et al. 2013; Nan et al. 2010) Let $\tilde{c} = \left\langle {(\underline{c} ,c,\bar{c});\omega_{{\tilde{c}}} ,u_{{\tilde{c}}} } \right\rangle$ be a TIFN, for any $0 \le \kappa \le \omega_{{\tilde{c}}}$ and $u_{{\tilde{c}}} \le \lambda \le 1$, where $0 \le \kappa + \lambda \le 1$, then $\tilde{c}_{\lambda }^{\kappa } = \tilde{c}^{\kappa } \cap \tilde{c}_{\lambda }$, that is $\tilde{c}_{\lambda }^{\kappa } = \tilde{c}^{\kappa } \cap \tilde{c}_{\lambda } = [\hbox{max} \{ \tilde{c}_{L}^{\kappa } ,\tilde{c}_{L}^{\lambda } \} ,\hbox{min} \{ \tilde{c}_{R}^{\kappa } ,\tilde{c}_{R}^{\lambda } \} ]$.

## Main results

### Problem description

This paper discusses a LCDS contract which guarantees a basket of housing mortgage loans. The borrower $B_{i} (i = 1,2 \ldots ,n)$ applies for a housing mortgage loan from bank A and repays both principal and regular monthly interest. Once a market interest rate is below the interest rate stipulated in the mortgage, the borrower may prepay the loan (such risk is defined as the risk of prepayment); when the house value falls and is lower than the amount remaining on the mortgage, the borrower will have reason to default. This presents the bank with a problem. To transfer these credit risks, bank A and investor C will sign a LCDS contract with the reference entity a basket of mortgages. In the contract, bank A is the credit protection buyer who pays a regular premium to C. Investor C is the credit protection seller who will reimburse A for the loss when a stated credit incident occurs during the period of the contract. If the reference entity is a mortgage, the loan repayment comes from two main sources: the first is the cash flow used for the repayment of the loan; the second one is the value of the mortgaged house when the reference entity defaults. Therefore, the compensation of the credit protection seller is related to the value of the mortgaged house in the first default. However, the financial market is changing. New financial policies, natural disasters, the current international and domestic political and economic outlook, and other factors, may affect house values. In addition, changes in house values are related to each other. Thus, it is more reasonable to describe the dynamic features using a one-factor copula function.^1^

#### **Definition 6**

(Li 2000) It is hypothesized that the factors that affect mortgage house values are of two kinds; systematic factors and non-systematic factors. Both kinds of factors are random and obey the standard normal distribution, and thus the $i$-th mortgage house value $V_{i}$ space (latent variable) can be expressed as the following one-factor gaussian copula model:

$$V_{i} = \sqrt {\rho_{i} } Y + \sqrt {1 - \rho_{i} } Z_{i} ,\quad i = 1,2, \ldots ,n.$$

where, systematic factor $Y$ stands for macroeconomic situations, non-systematic factor $Z_{i}^{\prime } s$ stands for a factor functional only single mortgage house asset $i$. And systematic factor $Y$ and non-systematic factor $Z_{i}^{\prime } s$ are mutually independent. The marginal distribution of mortgage house asset value $V_{i}^{\prime } s$ is conditionally independent under the condition that the systematic factor $Y$ is known. $\sqrt {\rho_{i} }$ is the correlation coefficient between mortgage house asset value and the systematic factor. Under the condition that both the systematic factor $Y$ and non-systematic factor $Z_{i}^{\prime } s$ obey the standard normal distribution, mortgage house asset value $V_{i}$ will also obey the standard normal distribution.

Methods in the literature (Rong et al. 2012) have improved and have adapted to describe the prepayment factors of housing mortgage loans. Hypothesize the borrower $B_{i} (i = 1, \ldots ,n)$ space applied to the bank for a housing loan $L_{0}^{i}$ at time zero, and $\sum\nolimits_{i = 1}^{n} {L_{0}^{i} } = K$. The borrower repays the loan at certain interest rate $\dot{r}$. The amount of repayment in unit time is $x$; the remaining loan amount $L^{i} (t,T)$ satisfies the following differential equation

$$\left\{ {\begin{array}{*{20}l} {\frac{{dL^{i} (t,T)}}{{dt}} = \dot{r}L^{i} (t,T) - x{\text{ 0 < t < T,}}} \hfill \\ {L^{i} (0,T) = L_{0}^{i} ,{\text{ }}L^{i} (T,T) = 0.} \hfill \\ \end{array} } \right.$$

which can be solved giving the result:

$$L^{i} (t,T) = L_{0}^{i} \frac{{e^{{\dot{r}T}} - e^{{\dot{r}t}} }}{{e^{{\dot{r}T}} - 1}}, \, x = L_{0}^{i} \frac{{\dot{r}e^{{\dot{r}T}} }}{{e^{{\dot{r}T}} - 1}}.$$

Prepayment is described as prepayment factor $Q_{t}^{i}$ which stands for the proportion of the actual residual loan remaining to the agreed residual loan remaining at time t. Therefore $0 \le Q_{t}^{i} \le 1$, $Q_{0}^{i} = 1$, and $Q_{t}^{i}$ is a diminishing process as time goes by. Nevertheless, the motivation of the borrower to prepay the loan is somewhat complex. It includes both market factors and factors particular to the borrower, such as changes in market rates of interest or changes in the financial position of the borrower. This may then result in a certain fuzziness and hesitation on the part of the credit protection buyer (the bank) relative to the prepayment of the borrower. Therefore, the changing intensity of $Q_{t}^{i}$ can be expressed with a reduced-form model as $\lambda_{t}^{i} = a_{i} r_{t} + b_{i} \beta_{t}^{i}$. Where, $a_{i}$ and $b_{i}$ are the sensitivity coefficients of the interest rate and the borrower’s own financial position with the hypothesis that the market interest rate $r_{t}$ and the borrower’s own peculiarities $\beta_{t}^{i}$ are mutually independent. One of the features of $Q_{t}^{i}$ is that the rate of change obeys ${{dQ_{t}^{i} }/{ { {Q_{t}^{i} }}} } = - \lambda_{t}^{i} dt$, resulting in $Q_{t}^{i} = e^{{ - \int_{0}^{t} {(a_{i} r_{t} + b_{i} \beta_{t}^{i} )dt} }}$, so the real loan amount remaining at time $t$ is

1 $$\bar{L}^{i} (t,T) = Q^{i} (t)L^{i} (t,T) = e^{{ - \int_{0}^{t} {(a_{i} r_{t} + b_{i} \beta_{t}^{i} )dt} }} L_{0}^{i} \frac{{e^{{\dot{r}T}} - e^{{\dot{r}t}} }}{{e^{{\dot{r}T}} - 1}}.$$

At the same time, to consider the influence of fuzziness and hesitation on LCDS pricing that objectively exists in the prepayment process, with the hypothesis that the sensitivity coefficients $a_{i}$ and $b_{i}$ in the reference mortgage loan prepayment change are triangle intuition based fuzzy numbers (to emphasize influence of the fuzziness and hesitation of the prepayment factor to LCDS pricing, this paper hypothesizes the correlation coefficient between mortgage house value and systematic factor $\sqrt {\rho_{i} }$ is a constant). That is

$$\tilde{a}_{i} =\,< (\underline{a}_{i} ,a_{i} ,\bar{a}_{i} );\omega_{{\tilde{a}_{i} }} ,u_{{\tilde{a}_{i} }} > ;\;\tilde{b}_{i} = < (\underline{b}_{i} ,b_{i} ,\bar{b}_{i} );\omega_{{\tilde{b}_{i} }} ,u_{{\tilde{b}_{i} }} > .$$

## The fuzzy form pricing model for LCDS

The first default pricing of a basket of LCDS is that if any mortgage loan in the reference mortgage loan pool defaults, the LCDS contract will be ended and credit protection seller C will pay the mortgage loan default loss to bank A. $\tau_{i} (i = 1,2, \ldots ,n)$ is expressed as the time of the *i*th reference mortgage loan default and $\tau = \min_{1 \le i \le n} \tau_{i}$ is the first default time in the reference mortgage loan (first default).

Experience overseas tells us that house prices can fall rapidly. When a house price falls to a certain level, the borrower will make a reasonable decision, based on economic principles, in his attempt to balance cost and profit. He may stop repayments on the amount of loan remaining when house value $V^{i} (t)$ is lower than the real amount of mortgage outstanding $\tilde{L}^{i} (t,T)$, (Merton 1974). Under the condition that systematic factor $Y = y$ is given, the $i$-th reference house mortgage conditional default probability is $(i = 1,2, \ldots ,n)$ as follow:

2 $$\begin{aligned} p_{t}^{i|Y} & = P \{ V_{i} \le \tilde{L}^{i} (t,T)|Y = y\} \\ & = P\{ \sqrt {\rho_{i} } Y + \sqrt {1 - \rho_{i} } Z_{i} \le \tilde{L}^{i} (t,T)|Y = y\} \\ & = P\left\{ {Z_{i} \le \frac{{\tilde{L}^{i} (t,T) - \sqrt {\rho_{i} } Y}}{{\sqrt {1 - \rho_{i} } }}|Y = y} \right\} \\ & = \Phi \left( {\frac{{\tilde{L}^{i} (t,T) - \sqrt {\rho_{i} } Y}}{{\sqrt {1 - \rho_{i} } }}} \right) \\ \end{aligned}$$

Using of the total expectation function (related concepts can be seen in reference Mukhopadhyay 2000), the probability of unconditional default can be put as:

3 $$\begin{aligned} p_{t}^{i} & = P(V_{i} \le \tilde{L}^{i} (t,T)) \\ & = E(1_{{\{ V_{i} \le \tilde{L}^{i} (t,T)\} }} ) \\ & = E(E(1_{{\{ V_{i} \le \tilde{L}^{i} (t,T)\} }} \left| Y \right. = y)) \\ & = E(P(V_{i} \le \tilde{L}^{i} (t,T)\left| {Y = y)} \right.) \\ & = E\left( {\Phi \left( {\frac{{\tilde{L}^{i} (t,T) - \sqrt {\rho_{i} } Y}}{{\sqrt {1 - \rho_{i} } }}} \right)} \right) \\ & = \int_{ - \infty }^{ + \infty } {\Phi \left( {\frac{{\tilde{L}^{i} (t,T) - \sqrt {\rho_{i} } Y}}{{\sqrt {1 - \rho_{i} } }}} \right)d\Phi (y)} \\ \end{aligned}$$

Thus, the unconditional survival probability of the *i*th reference mortgage loan is:

4 $$L(\tau_{i} > t) = 1 - \int_{ - \infty }^{ + \infty } {\Phi \left( {\frac{{\tilde{L}(t,T) - \sqrt {\rho_{i} } Y}}{{\sqrt {1 - \rho_{i} } }}} \right)d\Phi (y)} = 1 - p_{t}^{i} .$$

### **Theorem 1**

*The probabilities of a basket of LCDS contract reference mortgage loan without default and the first default are respectively*:

5 $$\tilde{L}(\tau > t) = \int_{ - \infty }^{ + \infty } {\mathop \Pi \limits_{i = 1}^{n} (1 - p_{t}^{i|Y} )d\Phi (y)} ;$$

6 $$\tilde{F}(\tau \le t) = \int_{ - \infty }^{ + \infty } {\mathop \Pi \limits_{\begin{subarray}{l} i = 1 \\ i \ne j \end{subarray} }^{n} (1 - p_{t}^{i|Y} )p_{t}^{j|Y} d\Phi (y)} .$$

### *Proof*

Using the qualities of conditional expectation, the joint survival probability of the whole reference mortgage loan agreement can be put as:

$$\begin{aligned} \tilde{L}(\tau > t) & = \tilde{L}(\mathop {\hbox{min} }\limits_{1 \le i \le n} \tau_{i} > t) \\ & = P(V_{1} > \tilde{L}^{1} (t,T),V_{2} > \tilde{L}^{2} (t,T), \ldots ,V_{n} > \tilde{L}^{n} (t,T)) \\ & = E(E(1_{{\{ V_{1} > \tilde{L}^{1} (t,T),V_{2} > \tilde{L}^{2} (t,T), \cdot \cdot \cdot ,V_{n} > \tilde{L}^{n} (t,T)\} }} \left| Y \right. = y)) \\ & = E(E(1_{{\{ V_{1} > \tilde{L}^{1} (t,T)\} }} \left| Y \right. = y) \cdot \cdot \cdot E(1_{{\{ V_{n} > \tilde{L}^{n} (t,T)\} }} \left| Y \right. = y)) \\ & = E\left( {\mathop \Pi \limits_{i = 1}^{n} (1 - p_{t}^{i|Y} )} \right) = \int_{ - \infty }^{ + \infty } {\mathop \Pi \limits_{i = 1}^{n} (1 - p_{t}^{i|Y} )d\Phi (y)} \\ \end{aligned}$$

which at the same time, hypothesizes the $j(j = 1,2, \ldots ,n)$ th mortgage loan first default at time $[t,t + dt]$.

As a result:

$$\begin{aligned} \tilde{F}(\tau \le t) & = P(\mathop {\hbox{min} }\limits_{1 \le i \le n} \tau_{i} \le t) = P(\tau_{1} > t, \ldots \tau_{j - 1} > t,\tau_{j} \le t + dt,\tau_{j + 1} > t, \ldots \tau_{n} > t) \\ & = E(E(1_{{\{ V_{1} > \tilde{L}^{1} (t,T), \ldots V_{j - 1} > \tilde{L}^{j - 1} (t,T),V_{j} \le \tilde{L}^{j} (t,T),V_{j + 1} > \tilde{L}^{j + 1} (t,T) \ldots ,V_{n} > \tilde{L}^{n} (t,T)\} }} \left| Y \right. = y)) \\ & = E(E(1_{{\{ V_{1} > \tilde{L}^{1} (t,T)\} }} \left| Y \right. = y) \cdot \cdot \cdot E(1_{{\{ V_{j - 1} > \tilde{L}^{j - 1} (t,T)\} }} \left| Y \right. = y)E(1_{{\{ V_{j} \le \tilde{L}^{j} (t,T)\} }} \left| Y \right. = y) \\ & \quad E(1_{{\{ V_{j + 1} > \tilde{L}^{j + 1} (t,T)\} }} \left| Y \right. = y) \cdot \cdot \cdot E(1_{{\{ V_{n} > \tilde{L}^{n} (t,T)\} }} \left| Y \right. = y)) \\ & = E(\mathop \Pi \limits_{\begin{subarray}{l} i = 1 \\ i \ne j \end{subarray} }^{n} (1 - p_{t}^{i|Y} )p_{t}^{j|Y} ) = \int_{ - \infty }^{ + \infty } {\mathop \Pi \limits_{\begin{subarray}{l} i = 1 \\ i \ne j \end{subarray} }^{n} (1 - p_{t}^{i|Y} )p_{t}^{j|Y} d\Phi (y)} \\ \end{aligned}$$

At that moment, the loss to The Bank is the real residual amount of principal of the $j$ th first default mortgage loan minus the house value is mortgaged, then the expectation of future cash flow discounted present value that the credit protection seller compensates to the protection buyer in a fuzzy environment is:

7 $$PV(loss) = E^{Q} \left[ {(\tilde{L}^{j} (\tau ,T) - V^{j} (\tau ))\exp \left( { - \int_{t}^{\tau } {r_{s} } ds} \right)1_{{\{ \tau \le T\} }} } \right],$$

Hypothesize the premium $\tilde{s}$ is paid at a discrete fixed time $0 < T_{1} < T_{2} \cdot \cdot \cdot < T_{n} = T$, then the expectation of future cash flow discounted to present value that the LCDS buyer pays to the LCDS seller in a fuzzy environment is:

8 $$PV(fee) = E^{Q} \left[ {\tilde{s}\sum\limits_{i = 1}^{n} {1_{{\{ \tau > T_{i - 1} \} }} (\tau \wedge T_{i} - T_{i - 1} )K\exp \left( - \int_{t}^{{\tau \wedge T_{i} }} {r_{s} } ds\right)} } \right],$$

*Q* is a risk neutral probability measure, $1_{{\{ \tau \le T\} }}$ is default indicative function. In other words, the function value is 1 when the first mortgage loan default happens in the reference mortgage loan pool, or the value is 0. According to the arbitrage-free pricing principle, initially $PV(loss) = PV(fee)$ so the first default fair premium of a basket of housing mortgage loan credit default swaps can be obtained.

### **Theorem 2**

*According to the arbitrage*-*free pricing principle, the fair premium of a basket of LCDS first default in a fuzzy environment is*:

9 $$\tilde{s} = \frac{{l_{1} }}{{(l_{2} + l_{3} )}},$$

*where,*

10 $$l_{1} = \frac{1}{{\sqrt {2\pi } }}\int_{t}^{T} {(\tilde{L}^{j} (u,T) - V^{j} (u))\mathop \Pi \limits_{\begin{subarray}{l} i = 1 \\ i \ne j \end{subarray} }^{n} (1 - p_{u}^{i|Y} )p_{u}^{j|Y} } \exp \left( { - \int_{t}^{u} {r_{s} ds - \frac{{u^{2} }}{2}} } \right)du;$$

11 $$l_{2} = K\sum\limits_{i = 1}^{n} {\Delta T_{i} \exp \left( { - \int_{t}^{{T_{i} }} {r_{u} du} } \right)\left( {\int_{ - \infty }^{ + \infty } {\mathop \Pi \limits_{i = 1}^{n} (1 - p_{u}^{i|Y} )d\Phi (y)} } \right)} ;$$

12 $$l_{3} = K\frac{1}{{\sqrt {2\pi } }}\sum\limits_{i = 1}^{n} {\int_{{T_{i - 1} }}^{{T_{i} }} {(u - T_{i - 1} )} \mathop \Pi \limits_{\begin{subarray}{l} i = 1 \\ i \ne j \end{subarray} }^{n} (1 - p_{u}^{i|Y} )p_{u}^{j|Y} \times \exp \left( { - \int_{t}^{u} {r_{s} ds - \frac{{u^{2} }}{2}} } \right)du} ;$$

13 $$\tilde{L}^{j} (t,T) = e^{{ - \int_{0}^{t} {(\tilde{a}_{i} r_{t} + \tilde{b}_{i} \beta_{t}^{j} )dt} }} L_{0}^{j} \frac{{e^{{\dot{r}T}} - e^{{\dot{r}t}} }}{{e^{{\dot{r}T}} - 1}}.$$

### *Proof*

On the basis of the probability of a basket of LCDS reference entity first default and the qualities of expectation:

$$\begin{aligned} PV(loss) & = E^{Q} \left[ {(\tilde{L}^{j} (\tau ,T) - V^{j} (\tau ))\exp \left( { - \int_{t}^{\tau } {r_{s} } ds} \right)1_{{\{ \tau \le T\} }} } \right] \\ & = \int_{t}^{T} {(\tilde{L}^{j} (u,T) - V^{j} (u))\exp \left( { - \int_{t}^{u} {r_{s} } ds} \right)d\tilde{F}(u)} \\ & = \frac{1}{{\sqrt {2\pi } }}\int_{t}^{T} {(\tilde{L}^{j} (u,T) - V^{j} (u))\mathop \Pi \limits_{\begin{subarray}{l} i = 1 \\ i \ne j \end{subarray} }^{n} (1 - p_{u}^{i|Y} )p_{u}^{j|Y} } \exp \left( { - \int_{t}^{u} {r_{s} ds - \frac{{u^{2} }}{2}} } \right)du \\ \end{aligned}$$

$$\begin{aligned} PV(fee) & = E^{Q} \left[ {\tilde{s}\sum\limits_{i = 1}^{n} {1_{{\{ \tau > T_{i - 1} \} }} } (\tau \wedge T{}_{i} - T_{i - 1} )K\exp \left( { - \int_{t}^{{\tau \wedge T_{i} }} {r_{s} } ds} \right)} \right] \\ & = \tilde{s}K\sum\limits_{i = 1}^{n} {\left[ {\int_{{T_{i - 1} }}^{{T_{i} }} {(u - T_{i - 1} )} \exp ( - \int_{t}^{u} {r_{s} } ds)d\tilde{F}(u) + \Delta T_{i} \exp ( - \int_{t}^{{T_{i} }} {r_{s} } ds)\tilde{L}(T_{i} )} \right]} \\ & = \tilde{s}K\sum\limits_{i = 1}^{n} {\Delta T_{i} } \exp \left( { - \int_{t}^{{T_{i} }} {r_{u} du} } \right)\left( {\int_{ - \infty }^{ + \infty } {\mathop \Pi \limits_{i = 1}^{n} (1 - p_{u}^{i|Y} )d\Phi (y)} } \right) + \tilde{s}K\frac{1}{{\sqrt {2\pi } }} \\ & \times \sum\limits_{i = 1}^{n} {\int_{{T_{i - 1} }}^{{T_{i} }} {(u - T_{i - 1} )} \mathop \Pi \limits_{\begin{subarray}{l} i = 1 \\ i \ne j \end{subarray} }^{n} \left( {1 - p_{u}^{i|Y} } \right)p_{u}^{j|Y} \times \exp \left( { - \int_{t}^{u} {r_{s} ds - \frac{{u^{2} }}{2}} } \right)du} \\ \end{aligned}$$

In conclusion, according to the arbitrage-free pricing principle, the theorem can be proved.

### Inference 1

On the basis of the definition of the cut set of TIFN, then the $\left\langle {\kappa ,\lambda } \right\rangle$-cut set of the fair premium of basket of LCDS first default $\tilde{s}$ is:

14 $$\tilde{s}_{\lambda }^{\kappa } = [\hbox{max} \{ \tilde{s}_{L}^{\kappa } ,\tilde{s}_{L}^{\lambda } \} ,\hbox{min} \{ \tilde{s}_{R}^{\kappa } ,\tilde{s}_{R}^{\lambda } \} ] ,$$

where,

$$\tilde{s}_{L}^{\kappa } = \left( {\underline{{\frac{{l_{1} }}{{(l_{2} + l_{3} )}}}} + \kappa \left( {\frac{{l_{1} }}{{(l_{2} + l_{3} )}} - \frac{{l_{1} }}{{(l_{2} + l_{3} )}}} \right)/\omega _{{\tilde{s}}} } \right);\;\tilde{s}_{R}^{\kappa } = \left( {\overline{{\frac{{l_{1} }}{{(l_{2} + l_{3} )}}}} - \kappa \left( {\overline{{\frac{{l_{1} }}{{(l_{2} + l_{3} )}}}} - \frac{{l_{1} }}{{(l_{2} + l_{3} )}}} \right)/\omega _{{\tilde{s}}} } \right)$$

$$\tilde{s}_{L}^{\lambda } = \left[ {(1 - \lambda )\frac{{l_{1} }}{{(l_{2} + l_{3} )}} + (\lambda - u_{{\tilde{s}}} )\underline{{\frac{{l_{1} }}{{(l_{2} + l_{3} )}}}} } \right]/(1 - u_{{\tilde{s}}} );\;\tilde{s}_{R}^{\lambda } = \left[ {(1 - \lambda )\frac{{l_{1} }}{{(l_{2} + l_{3} )}} + (\lambda - u_{{\tilde{s}}} )\overline{{\frac{{l_{1} }}{{(l_{2} + l_{3} )}}}} } \right]/(1 - u_{{\tilde{s}}} )$$

$$\underline{{\tilde{s}}} = \underline{{\left[ {\frac{{l_{1} }}{{(l_{2} + l_{3} )}}} \right]}} = \frac{{\underline{{l_{1} }} }}{{(\bar{l}_{2} + \bar{l}_{3} )}},\;\bar{\tilde{s}} = \left[ {\overline{{\frac{{l_{1} }}{{(l_{2} + l_{3} )}}}} } \right] = \frac{{\bar{l}_{1} }}{{(\underline{{l_{2} }} + \underline{{l_{3} }} )}},$$

$$\underline{{l_{1} }} = \frac{1}{{\sqrt {2\pi } }}\int_{t}^{T} {(\underline{{\tilde{L}^{j} (u,T)}} - V^{j} (u))\mathop \Pi \limits_{\begin{subarray}{l} i = 1 \\ i \ne j \end{subarray} }^{n} (1 - \overline{{p_{u}^{i|Y} }} )\underline{{p_{u}^{j|Y} }} } \exp \left( { - \int_{t}^{u} {r_{s} ds - \frac{{u^{2} }}{2}} } \right)du,$$

$$\bar{l}_{1} = \frac{1}{{\sqrt {2\pi } }}\int_{t}^{T} {(\overline{{\tilde{L}^{j} (u,T)}} - V^{j} (u))\mathop \Pi \limits_{\begin{subarray}{l} i = 1 \\ i \ne j \end{subarray} }^{n} (1 - \underline{{p_{u}^{i|Y} }} )\overline{{p_{u}^{j|Y} }} } \exp \left( { - \int_{t}^{u} {r_{s} ds - \frac{{u^{2} }}{2}} } \right)du,$$

$$\underline{{l_{2} }} = K\sum\limits_{i = 1}^{n} {\Delta T_{i} \exp \left( { - \int_{t}^{{T_{i} }} {r_{u} du} } \right)\left( {\int_{ - \infty }^{ + \infty } {\mathop \Pi \limits_{i = 1}^{n} (1 - \overline{{p_{u}^{i|Y} }} )d\underline{\Phi (y)} } } \right)} ,$$

$$\bar{l}_{2} = K\sum\limits_{i = 1}^{n} {\Delta T_{i} \exp \left( { - \int_{t}^{{T_{i} }} {r_{u} du} } \right)\left( {\int_{ - \infty }^{ + \infty } {\mathop \Pi \limits_{i = 1}^{n} (1 - \underline{{p_{u}^{i|Y} }} )d\overline{\Phi (y)} } } \right)} ,$$

$$\underline{{l_{3} }} = K\frac{1}{{\sqrt {2\pi } }}\sum\limits_{i = 1}^{n} {\int_{{T_{i - 1} }}^{{T_{i} }} {(u - T_{i - 1} )} \mathop \Pi \limits_{\begin{subarray}{l} i = 1 \\ i \ne j \end{subarray} }^{n} (1 - \overline{{p_{u}^{i|Y} }} )\underline{{p_{u}^{j|Y} }} \times \exp \left( { - \int_{t}^{u} {r_{s} ds - \frac{{u^{2} }}{2}} } \right)du} ,$$

$$\bar{l}_{3} = K\frac{1}{{\sqrt {2\pi } }}\sum\limits_{i = 1}^{n} {\int_{{T_{i - 1} }}^{{T_{i} }} {(u - T_{i - 1} )} \mathop \Pi \limits_{\begin{subarray}{l} i = 1 \\ i \ne j \end{subarray} }^{n} (1 - \underline{{p_{u}^{i|Y} }} )\overline{{p_{u}^{j|Y} }} \times \exp \left( { - \int_{t}^{u} {r_{s} ds - \frac{{u^{2} }}{2}} } \right)du} ,$$

$$\underline{{\tilde{L}^{j} (u,T)}} = e^{{ - \int_{0}^{t} {(\bar{a}_{i} r_{t} + \bar{b}_{i} \beta_{t}^{j} )dt} }} L_{0}^{j} \frac{{e^{{\dot{r}T}} - e^{{\dot{r}t}} }}{{e^{{\dot{r}T}} - 1}},\;\overline{{\tilde{L}^{j} (u,T)}} = e^{{ - \int_{0}^{t} {(\underline{{a_{i} }} r_{t} + \underline{{b_{i} }} \beta_{t}^{j} )dt} }} L_{0}^{j} \frac{{e^{{\dot{r}T}} - e^{{\dot{r}t}} }}{{e^{{\dot{r}T}} - 1}}.$$

### *Proof*

The proof can be made by the definition of TIFN’s and the monotonic property of the function.

## Numerical analysis

Hypothesize that the sensitivity coefficient of the prepayment change intensity and market interest rate is:

$$\tilde{a}_{j} = \left\langle {(0.9,1.1,1.2);0.6,0.3} \right\rangle ,$$

Thus, the possibility that the sensitivity coefficient of the motivation of the borrower’s prepayment the investor thought to the market interest rate is 1.1 will be 0.6. By the same token, the impossibility of it will be 0.3, and the hesitation degree will be 0.1. If the sensitivity coefficient of prepayment and the borrower’s own particular circumstances are:

$$\tilde{b}_{j} = \left\langle {(0.7,0.8,1.0);0.7,0.2} \right\rangle ,$$

then the possibility that the sensitivity coefficient of the motivation of borrower’s prepayment the investor thought to the borrower’s own features is 0.8 will be 0.7. Thus, the impossibility of it will be 0.2, and the hesitation degree will be 0.1.

Other relevant parameters are set as follows, $r_{t} = 0.06$, $\beta_{t}^{j} = 0.03$, $\dot{r} = 0.07$, $n = 1$, $L_{0}^{j} = 1$, $K = 1$, $Y = 1$, $\rho_{j} = 0.4$. Therefore, according to Theorem 2 and Inference 1, the $\left\langle {\kappa ,\lambda } \right\rangle$-cut set of the first default price of LCDS in the most active overseas market 5 year period can be obtained through MATLAB analog computing.

**Fig. 1:**
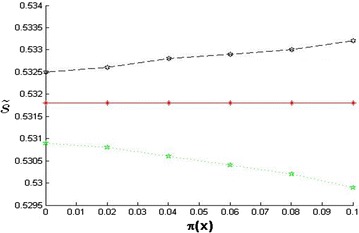
The dynamic relationship between hesitation degree and fuzzy price of the first default of LCDS (k = 0.3, λ = 0.6)

From Table 1, we see that, as $k$ increases and $\lambda$ decreases, the fuzzy price range of the first default of a basket of LCDS gradually gets smaller. That means the investor may get a smaller price range by choosing a bigger $k$ and a smaller $\lambda$. From Fig. 1, it can be postulated that when the values of $k$ and $\lambda$ are fixed, an increase in an investor’s hesitation causes the fuzzy price range of the first default of basket of LCDS to get bigger. That means the investor can more accurately predict the price of LCDS in a fuzzy environment by decreasing the degree of hesitation (the horizontal line in Fig. 1 indicates no consideration of LCDS price in a fuzzy environment). Thus, when the investor is fuzzy about and hesitant on the prepayment risk of the reference mortgage loan, to introduce a fair premium LCDS with a triangular intuition-based fuzzy number is more like the real market environment. When compared to models in the existing literature (Wu and Liang 2011; Rong et al. 2012; Liang et al. 2013; Zhou and Liang 2010; Wu 2012; Liang and Wang 2012; Wang et al. 2012), our new fuzzy environment LCDS pricing model can consider more default influence factors, can more closely resemble the complexities of the dynamics of default, can employ the membership function and the non-membership function to simultaneously describe the fuzzy phenomenon, can enable the fuzzy phenomenon to be estimated in three kinds of state (the possible degree, the impossible degree and the hesitation degree), and can simultaneously reflect fuzziness and the hesitation in financial markets.

**Table 1:** Fuzzy prices of the first default of LCDS in different cut set levels

$\kappa$	0	0.1	0.2	0.3	0.4	0.5
$\lambda$	1	0.9	0.7	0.6	0.6	0.5
$T = 5$	[0.5275, 0.5352]	[0.5282, 0.5346]	[0.5293, 0.5337]	[0.5299, 0.5332]	[0.5304, 0.5329]	[0.5311, 0.5324]

## Conclusions

Housing mortgage loan credit default swaps are powerful instruments used to transfer and hedge housing mortgage loan credit risk. However, the contingency of the financial market and the incompleteness of information will cause fuzziness and hesitation on the part of investors when it comes to the prepayment of a mortgage loan in question. This paper therefore introduces triangular intuition-based fuzzy numbers into LCDS pricing models in order to describe the fuzziness and hesitation surrounding prepayments. It also analyzes the sensitivity of that hesitation, and finds a model that considers the fair price of LCDS in a fuzzy random environment, including a pure random environment. A proper price range offers the investor more flexible options and also makes the model more closely resemble the real market environment. Thus, this paper to a certain extent puts forward a creative and practical model as a new LCDS pricing instrument for the financial practitioner and offers a brand new theoretical basis to transfer and hedge the credit risk of housing mortgage loans. Of course, there are some deficiencies in this paper. For example, due to limited market data, we did not check the model parameters for market data, but this we will study in the future. Meanwhile, because the financial market structure in China is less flexible, it is hard to disperse and configure the risks, especially the credit risks which are difficult to measure. This means that there is more fuzziness in the pricing environment and more asymmetric information between financial institutions with a concentration of risks and investors. Furthermore, the attitude of investors towards fuzzy information is so diversified that it is difficult to estimate default situations; fuzzy pricing for other credit derivatives will also be the focus of our research in the future.

## Footnotes

1 The key of a basket of LCDS pricing is to describe the default correlation of different reference mortgage loans. Moreover, one-factor copula function is superior in building the joint probability of default in a combination of credit assets pricing model—it is simple, convenient to calculate and easy to extend.
